# Persistent Submicroscopic Plasmodium falciparum Parasitemia 72 Hours after Treatment with Artemether-Lumefantrine Predicts 42-Day Treatment Failure in Mali and Burkina Faso

**DOI:** 10.1128/AAC.00873-21

**Published:** 2021-07-16

**Authors:** Khalid B. Beshir, Nouhoum Diallo, Fabrice A. Somé, Salif Sombie, Issaka Zongo, Bakary Fofana, Aliou Traore, Souleymane Dama, Amadou Bamadio, Oumar B. Traore, Sam A. Coulibaly, Ouattara S. Maurice, Amidou Diarra, Jean Moise Kaboré, Aly Kodio, Amadou Hamidou Togo, Niawanlou Dara, Moctar Coulibaly, Francois Dao, Frederic Nikiema, Yves D. Compaore, Naomie T. Kabore, Nouhoun Barry, Issiaka Soulama, Issaka Sagara, Sodiomon B. Sirima, Jean-Bosco Ouédraogo, Abdoulaye Djimde, Colin J. Sutherland

**Affiliations:** a Faculty of Infectious and Tropical Diseases, London School of Hygiene and Tropical Medicine, London, United Kingdom; b Malaria Research and Training Centre, Department of Epidemiology of Parasitic Diseases, Faculty of Pharmacy, University of Sciences, Techniques and Technologies of Bamako, Bamako, Mali; c Institut de Recherche en Sciences de la Santé Direction Régionale de l’Ouest, Bobo-Dioulasso, Burkina Faso; d Centre National de Recherche et de Formation sur le Paludisme, Ougadougou, Burkina Faso; e Groupe de Recherche Action en Santé, Ougadougou, Burkina Faso

**Keywords:** *Plasmodium falciparum*, antimalarial agents

## Abstract

A recent randomized controlled trial, the WANECAM (West African Network for Clinical Trials of Antimalarial Drugs) trial, conducted at seven centers in West Africa, found that artemether-lumefantrine, artesunate-amodiaquine, pyronaridine-artesunate, and dihydroartemisinin-piperaquine all displayed good efficacy. However, artemether-lumefantrine was associated with a shorter interval between clinical episodes than the other regimens. In a further comparison of these therapies, we identified cases of persisting submicroscopic parasitemia by quantitative PCR (qPCR) at 72 h posttreatment among WANECAM participants from 5 sites in Mali and Burkina Faso, and we compared treatment outcomes for this group to those with complete parasite clearance by 72 h. Among 552 evaluable patients, 17.7% had qPCR-detectable parasitemia at 72 h during their first treatment episode. This proportion varied among sites, reflecting differences in malaria transmission intensity, but did not differ among pooled drug treatment groups. However, patients who received artemether-lumefantrine and were qPCR positive at 72 h were significantly more likely to have microscopically detectable recurrent Plasmodium falciparum parasitemia by day 42 than those receiving other regimens and experienced, on average, a shorter interval before the next clinical episode. Haplotypes of *pfcrt* and *pfmdr1* were also evaluated in persisting parasites. These data identify a possible threat to the parasitological efficacy of artemether-lumefantrine in West Africa, over a decade since it was first introduced on a large scale.

## INTRODUCTION

The antimalarial efficacy of artemisinin-based combination therapies (ACT) against Plasmodium falciparum infections in Africa remains high, with >90% efficacy for all current regimens (1). Despite this generally positive picture, evidence is growing that parasites with reduced susceptibility do occur across the continent. In the latest *World Malaria Report*, the WHO notes four studies in Malawi, Angola, Uganda, and The Gambia in which more than 10% of malaria patients treated with the widely deployed ACT artemether-lumefantrine (AL) failed treatment (2). Similar indications were evident in recent clinical trials. The multicenter WANECAM (West African Network for Clinical Trials of Antimalarial Drugs) study treated 4,710 individuals for clinical malaria in Burkina Faso, Guinea, and Mali and followed them for 2 years, finding that both established and recently licensed ACT provided PCR-corrected adequate clinical and parasitological response (ACPR) against Plasmodium falciparum malaria in all 7 study sites (3). However, AL did not perform as well as either of the newer formulations pyronaridine-artesunate (PA) and dihydroartemisinin-piperaquine (DP) as indicated by, first, lower efficacy (uncorrected) at 28 and 42 days after treatment and, second, a shorter time to next malaria episode than in patients randomized to receive either DP or PA. Both observations may be attributable to the shorter elimination half-life of lumefantrine (4 days) than of pyronaridine (13 days) and piperaquine (4 weeks) (1). Nevertheless, posttreatment parasite isolates from a subset of AL-treated Malian patients in the WANECAM cohort demonstrated a higher 50% effective concentration (EC_50_) than did pretreatment isolates for both artemether and lumefantrine in *ex vivo* susceptibility testing (4), suggesting a waning parasite susceptibility to AL consistent with other findings in West Africa (5, 6). Relevant findings also come from the other side of the continent; our Kenyan studies found that subpatent parasite persistence in AL- and DP-treated patients was strongly linked to subsequent microscopically detectable parasite recrudescence 4 to 6 weeks later (7) and found significant selection for an Asn substitution at codon 86 of *pfmdr1* (8), the locus encoding the parasite digestive vacuole (DV) membrane transporter PgH1. Further, recent studies of imported P. falciparum malaria cases in Sweden and the United Kingdom document cases of AL treatment failure in parasites of African origin (9, 10). Therefore, there is an urgent need to examine the efficacy of AL in African settings and test for association with resistance-associated markers in order to inform molecular surveillance efforts.

The determinants of parasite susceptibility to lumefantrine remain unclear, and *in vitro* correlates of susceptibility are not yet well established. Cultured parasite isolates from patients that have failed AL treatment display little difference in standard lumefantrine EC_50_ estimates from those of isolates from successfully treated patients (11). However, it has been established that the wild-type codon 86 Asn (N) allele in *pfmdr1* has a strong selective advantage over the chloroquine-selected 86 Tyr (Y) mutation, with 86N showing significant directional selection in a number of African studies of AL-treated malaria patients (8, 12–14). A statistically significant increase in lumefantrine EC_50_ is also seen when the 86N allele replaces the Y allele in gene-editing experiments in chloroquine-resistant lab-adapted parasite strains (15). Nevertheless, there is as yet no compelling evidence that this change in *pfmdr1* alone is sufficient to engender full-blown lumefantrine resistance *in vivo*, and there are compelling reasons to believe that AL also exerts selective pressure on the *pfcrt* locus (8), which encodes another transporter protein in the parasite digestive vacuole membrane and is the key determinant of chloroquine and amodiaquine susceptibility (16, 17).

Over the last decade, a clearer picture of the key determinants of susceptibility to the artemisinin component of ACT has emerged, relevant to the emergence in Southeast Asia of P. falciparum harboring certain variants of the Kelch propeller domain protein K13, encoded by the *pfk13* gene. Patients infected with these parasites display slower parasite clearance after treatment with either artemisinin monotherapy or ACT (18–21), and *in vitro* correlates have been developed which demonstrated that any one of these specific mutations is both necessary and sufficient to enhance parasite survival against a short pulse of high-dose artemisinin *in vitro* (22, 23). Surveillance of *pfk13* genotypes across Africa has been carried out since the identification of this resistance-associated locus, but to date, the key mutations associated with reduced artemisinin susceptibility in Southeast Asia have been absent in population surveys (24–27) and in sequence analyses of African parasite isolates from patients who failed treatment with AL (5, 6, 28). Recently, a focus of P. falciparum harboring the R561H propeller domain variant of *pfk13* was identified in Rwanda (29). This genotype was associated with both slow parasite clearance and parasitological treatment failure at day 28. Further work is required to ascertain whether these parasites represent a growing threat to the clinical efficacy of AL in east Africa.

Evaluation of clinical efficacy in antimalarial drug trials would be greatly advanced by efficient and sensitive quantitative molecular methods to establish posttreatment parasite clearance dynamics and to detect parasites persisting during the posttreatment follow-up period. In this study, we deployed quantitative PCR (qPCR) amplification of parasite DNA from the WANECAM study. PCR-corrected drug efficacy findings for this study of 4,710 ACT-treated participants, carried out between October 2011 and December 2013, have been previously published (3). We present new parasite clearance data for a subset of 552 participants to explicitly explore the relationship between parasite persistence in the first 72 h following initiation of treatment and three outcomes from the main study: (i) uncorrected ACPR during 42 days of active follow-up, (ii) time to the next episode of symptomatic malaria over 2 years of passive follow-up, and (iii) microscopic gametocyte carriage at day 7.

In patients with persisting submicroscopic parasites at day 3, we also tested for evidence of directional selection at the *pfcrt* and *pfmdr1* loci.

## RESULTS

Baseline information for all 552 individuals analyzed, including treatment group in the parent randomized trial, is given in Table 1. Of these, 280 experienced a second malaria episode; blood samples at all four time points were available for 276. These second episodes occurred a median of 89 days after episode 1 (interquartile range, 60 to 201 days) among the 241 individuals for which both dates were available. Equal numbers of participants evaluated in this subanalysis received the two investigational regimens PA and DP (178 and 177, respectively). These were compared to standard ACT for the site concerned: 126 participants received AL and 71 received artesunate-amodiaquine (ASAQ), which was used in two sites only (Table 1). For the following analyses, individuals in the ASAQ treatment arm contributed to each combined analysis but were not included in stratified statistical tests, as participants were not randomized between the AL and ASAQ regimens (1).

**TABLE 1:** Baseline information for all 552 patients contributing to the analysis

Site	% in treatment group^*a*^				Proportion under 5 yrs	Proportion with episode 2^*b*^	Proportion female
	PA (*n* = 172)	AL (*n* = 121)	DP (*n* = 176)	ASAQ (*n* = 71)			
Sotuba (*n* = 128)	27.3	47.7	25.0		6.2	39.7	22.7
Kolle (*n* = 105)	33.3	33.3	33.3		14.3	78.1	43.8
Bougoula Hameau (*n* = 105)	31.4		34.3	34.3	10.1	82.7	53.7
Ninagoloko (*n* = 105)	33.3		33.3	33.3	19.8	32.1	55.3
Bobo-Dioulasso (*n* = 109)	36.7	27.5	35.8		19.3	25.9	52.9
ACPR (%) (uncorrected)	87.8	80.1	97.2	76.6			

a PA and DP, the investigational regimens, were tested in all sites against either one of the two comparator regimens, AL and ASAQ (artesunate-amodiaquine).

b Episode 2 is a subsequent clinical malaria episode diagnosed at least 28 days after the primary episode in the same individual. A total of 280 individuals in the analysis experienced a second episode. The same study drug was always given (1). Study site staff decided which individuals to include in this analysis; it was not a random selection, and therefore, the proportion of 2nd episodes in each site has no epidemiological significance.

### Parasite clearance dynamics in the first 72 h of treatment.

Extracted DNA from all time points up to day 3 was analyzed by duplex qPCR, with each time point run twice. A total of 405 treated participants at episode 1 were qPCR negative by day 2 (73.4%), and a further 64 participants displayed a relative parasite density between 0 and 0.1% of that on day 0; all 469 met the criteria for good clearance (parasite reduction rate at 48 h [PRR_48h_] estimate of 1,000 or greater). For the remaining 83 infections classed as slow clearing for episode 1 (15.0%), day 2 parasite density ranged from 0.11% to 92.1% of day 0 parasite density (median, 0.69%; interquartile range, 0.21% to 2.8% of day 0 parasite density). Thirty-eight of 276 evaluable infections in episode 2 were slow clearing (13.8%), with day 2 parasite density ranging from 0.11% to 155.8% of day 0 density (median, 0.56%; interquartile range, 0.28% to 3.68%).

**FIG 1:**
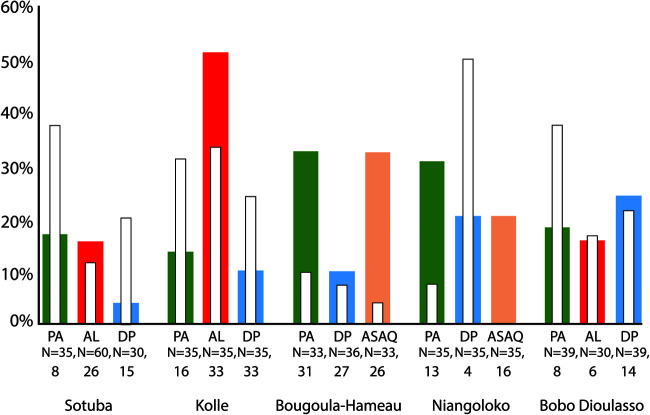
Proportion of participants qPCR positive at day 3 for episodes 1 and 2. Colored bars represent proportion of slow-clearing P. falciparum infections for each of the 4 treatment groups in the primary malaria episode, and inlaid white bars represent proportion for episode 2 in the same patient group. Denominators are given for episode 1 and episode 2.

Two binary parasite clearance parameters were derived from the analysis: slow clearance, defined as PRR_48h_ of less than 1,000, and day 3 qPCR positivity. Within patient groups defined by treatment arm and study site, there was no general pattern of correlation between episode 1 and episode 2 in either clearance parameter; data for day 3 qPCR positivity in both episodes are presented in Fig. 1. For episode 1, parasitemia was cleared more slowly (i.e., PRR_48h_ was more often below 1,000) with AL than with PA or DP among patients recruited at Kolle (odds ratio [OR], 4.5, 95% confidence interval [CI], 1.20 to 18.50; *P* = 0.008), but this difference was not seen among those recruited at Sotuba or Bobo Dioulasso. Similarly, only in Kolle was there a substantially higher prevalence of qPCR-positive day 3 parasitemia in the AL group than in the PA and DP groups (OR, 6.75; 95% CI, 2.18 to 22.1; *P* < 0.001). In paired analyses on an individual level, slow clearance in episode 1 was not associated with a higher chance of slow clearance occurring in episode 2 (OR, 0.850; 95% CI, 0.243 to 2.40; *P* = 0.75), and being qPCR positive on day 3 in episode 1 was not associated with a higher chance of being qPCR positive on day 3 in episode 2 (OR, 0.674; 95% CI, 0.240 to 1.65; *P* = 0.369).

The detection of persistent parasite carriage over the first 72 h posttreatment could plausibly be associated with higher parasite densities at enrollment, which may take longer to clear. We therefore compared parasite densities by microscopy (as previously reported [3]) for individuals found to be qPCR positive at day 3 with those from individuals that were qPCR negative at day 3. Among all 470 evaluable individuals with episode 1 parasite density available, those found to be qPCR positive at day 3 displayed higher day 0 parasite densities (*P* = 0.002; Wilcoxon rank sum test). However, after stratification by treatment arm, this relationship was maintained for ASAQ (*P* = 0.039; *n* = 53) and PA (*P* = 0.042; *n* = 152) but not for AL (*P* = 0.175; *n* = 124) or DP (*P* = 0.570; *n* = 141).

### Parasite genotypes in day 3 positives.

**FIG 2:**
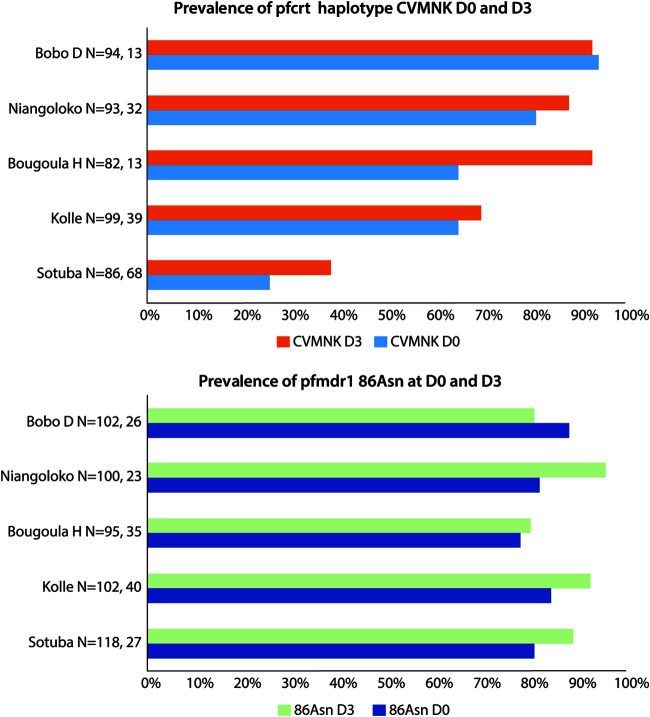
Prevalence of *pfcrt* CVMNK and *pfmdr1* codon 86Asn alleles among day 0 and day 3 isolates by study site. Denominators shown are for day 0 and day 3.

Previous studies suggest that ACT, and artemisinin in particular, selects against the *pfmdr1* 86 Tyr and *pfcrt* 72 to 76 CVIET genotypes that had previously been favored by the use of chloroquine (4, 8, 12–15). The prevalence of the corresponding wild-type alleles *pfmdr1* 86 Asn and *pfcrt* 72 to 76 CVMNK are shown in Fig. 2, for each study site, at day 0 and day 3 of episode 1. *pfcrt* genotyping was successful in 40.2% of qPCR positives from the parasite clearance data and in 22.9% of the negatives. *pfmdr1* sequencing was successful in 47.4% of the positives and 19.0% of the negatives. Pretreatment prevalence of CVMNK varied among the 5 sites but appeared to increase in prevalence in most sites at day 3 (Fig. 2, upper graph). *pfmdr1* 86 Asn was at 80% or higher prevalence at day 0 in all sites, and increases (4 sites) or decreases (1 site) by day 3 were small (Fig. 2, lower graph). As an exploratory paired analysis, we tested the full day 3 data set for evidence of selection against CVIET and *pfmdr1* 86 Tyr within each individual infection. Perhaps reflecting the already low prevalence at day 0, there was no evidence of significant directional selection against *pfmdr1* 86Tyr (*n* = 149; *P* = 0.086), but in contrast, we did find evidence of directional selection against *pfcrt* 72 to 76 CVIET (*n* = 137; *P* = 0.008).

### Association between parasite clearance and treatment outcome.

The first of two primary outcome measures in the main clinical study was the incidence density of malaria episodes over 2 years of follow-up. We therefore tested for evidence that slow clearance or day 3 qPCR positivity after treatment for episode 1 was associated with a shorter interval between episode 1 and episode 2 among the 280 individuals for whom we had paired assessments. The distribution of recorded times between episode 1 and episode 2 was found to fail the Shapiro-Wilks test for normality both with and without lognormal transformation, so nonparametric methods were used to evaluate association with this variable. By the Wilcoxon rank sum test, weak evidence was found across all 233 evaluable individuals that a shorter interval to episode 2 was associated with qPCR day 3 positivity but was not associated with slow clearance, defined as PRR_48h_ below 1,000 (Table 2). Stratifying by treatment arm, we found that the association between qPCR day 3 positivity and shorter interval to episode 2 was entirely resident in the AL-treated participants and was not found among participants treated with PA or DP (Table 2).

**TABLE 2:** Univariate associations between parasite clearance and treatment outcomes^*a*^

Parameter	Treatment arm	Shorter time to E2^*b*^	OR (95% CI) for E1 day 42 ACPR not achieved	OR (95% CI) for gametocytes at day 7
Slow clearance (PRR_48h_ < 1,000)	All combined^*c*^	83 days vs 91 days (*n* = 241; *P* = 0.484)	1.185 (0.427–2.85) (*n* = 536; *P *= 0.696)	**4.178 (0.977–16.8) (*n* = 450; *P* = 0.013)**
	PA			**9.92 (0.490–590) (*n* = 146; *P* = 0.025)**
	AL			0.0 (*n* = 124; *P* = 0.660)
	DP			4.7 (0.365–43.9) (*n* = 143; *P* = 0.073)
qPCR positive day 3	All combined^*c*^	**66 vs 95 days (*n* = 45, 188; *P* = 0.0032)**	**2.69 (1.24–5.58) (*n* = 536; *P* = 0.004)**	0.820 (0.085–4.06) (*n* = 453; *P *= 0.802)
	PA	76 vs 110 days (*n* = 13, 52; *P* = 0.549)	2.58 (0.525–10.4) (*n* = 172; *P* = 0.133)	
	AL	**43 vs 84 days (*n* = 17, 52; *P* = 0.0043)**	**5.23 (1.44–19.0) (*n* = 121; *P* = 0.002)**	
	DP	133 vs 98 days (*n* = 7, 63; *P* = 0.899)	0.0 (0.0–7.17) (*n* = 174; *P* = 0.453)	

a E1, treated clinical malaria episode 1; E2, treated clinical malaria episode 2.

b Measured in days; for significant differences (bold), median number of days is shown for the 2 groups. Accurate dates for E2 were not available for some individuals; ASAQ-treated individuals (*n* = 29) were retained in the combined analysis but are not shown in the stratification.

c Stratification by treatment arm is presented only where an effect was seen in the combined analysis. Stratified analysis for ASAQ-treated patients (*n* = 29) is not shown.

The second primary outcome of the parent clinical trial that we examined was uncorrected ACPR over 28 or 42 days of follow-up (1). We therefore tested for evidence that slow clearance or day 3 qPCR positivity was associated with treatment failure (i.e., recurrent parasitemia by microscopy) on or before day of episode 1 among 536 evaluable participants. In the combined data set, slow clearance (i.e., PRR_48h_ of <1,000 by qPCR) was not a risk factor for subsequent treatment failure among these participants; in contrast, qPCR positivity at day 3 was significantly associated with treatment failure on or before day 42 (Table 2). When this analysis was stratified by treatment group, failure to reach ACPR at day 42 was strongly associated with qPCR positivity at day 3 in the AL-treated group but was not associated in the PA-treated group or the DP-treated group (Table 2).

Finally, we examined the relationship between slow clearance and qPCR positivity at day 3 and microscopically detected P. falciparum gametocytes at day 7, as in our previous Kenyan studies (3). Evidence of an association between slow clearance and gametocyte carriage was found among all evaluable individuals. Interestingly, after stratification, this association was strong among PA-treated participants and borderline nonsignificant among DP-treated individuals but absent in the AL-treated group (Table 2). There was no association between day 7 gametocyte carriage and qPCR positivity at day 3.

### Factors affecting the association between day 3 positivity and ACPR.

The analyses presented in Table 2 suggest a relationship between qPCR positivity at day 3 and treatment failure, defined as not exhibiting ACPR by day 42. However, there are a number of potential confounders generally accepted as affecting the likelihood of ACPR, the principal example being patient age. Other key covariates that may impact this association include initial parasite density, sex, study site, and treatment arm. To examine the impact of these factors on the OR of the association between qPCR positivity on day 3 and day 42 ACPR, we used logistic regression. As shown in Table 3, the OR of 2.70 (1.24 to 5.58) from Table 2 fell to 2.11 but remained significant in a simple model including only patient age. When age, log-transformed parasite density, sex, study site, and treatment arm are all included in the model, there is little further change in the OR, with the association remaining significant and no increase in standard error of the estimate. We conclude that our finding of an association between qPCR positivity at day 3 and treatment failure remains robust after correction for potential confounding by age, parasite density, sex, study site, and treatment arm.

**TABLE 3:** Logistic regression of potential confounding factors for the association between qPCR positivity at day 3 and failure to achieve ACPR at day 42 in episode 1 in all drug arms

Model no.	Age	Day 0 parasite density (ln)^*b*^	Sex	Study site	Treatment arm	OR (95% CI)	SE	*P*
1 (*n* = 536)						2.70 (1.53–4.76)	0.782	0.001
2 (*n* = 476^*a*^)	X					2.11 (1.11–4.03)	0.695	0.023
3 (*n* = 464^*b*^)		X				2.05 (1.08–3.90)	0.674	0.013
4 (*n* = 464)	X	X				1.95 (1.01–3.73)	0.646	0.045
5 (*n* = 464)	X	X	X			1.98 (1.03–3.80)	0.659	0.041
6 (*n* = 464)	X	X	X	X		2.02 (1.05–3.89)	0.676	0.035
7 (*n* = 464)	X	X	X		X	1.97 (1.03–3.80)	0.659	0.042
8 (*n* = 464)	X	X	X	X	X	2.02 (1.05–3.89)	0.675	0.036

a Age data were missing for 60 individuals.

b Regression was performed against log-transformed microscopy-determined parasite densities (1). Day 0 parasite density was missing for 62 individuals.

## DISCUSSION

In this study, we used qPCR to estimate two parameters related to parasite clearance dynamics in over 800 episodes of falciparum malaria experienced by 552 study participants treated with ACT in Mali and Burkina Faso. In particular, we sought evidence that AL was inferior to DP or PA in initial clearance of parasitemia up to day 3 and whether this was associated with three key outcomes: time to next malaria episode, uncorrected ACPR at day 42, and microscopically detectable gametocyte carriage at day 7. In one site, Kolle, there was some evidence that slow clearance of parasitemia and qPCR positivity at day 3 was higher in the AL-treated group than for PA or DP, but this was seen only for episode 1, as parasite clearances were similar for PA and AL among the 82 second episodes from Kolle in our sample set and only slightly better for DP (Fig. 1 and 2). However, parasite clearance did have an impact on outcomes, as those that were qPCR positive at day 3 experienced a significantly shorter period before a subsequent malaria episode (a median of 66 versus 95 days) and were more likely to fail treatment by day 42, as evidenced by microscopically detected parasitemia during study follow-up. After stratification, both of these associations remained significant only in the AL-treated group. Gametocyte carriage at day 7 was weakly associated with slow clearance, but AL performed better than PA or DP. We conclude that in the PA and DP treatment groups, qPCR-detected parasite persistence immediately after treatment was related to gametocyte emergence in many instances, whereas in the group treated with AL, which has an impact on gametocyte carriage superior to those of other ACT (1, 3, 30, 31), parasite persistence was more likely indicative of persisting asexual parasitemia. We also show that in settings where the chloroquine-resistant alleles of *pfcrt* are still present (Fig. 2), artemisinin treatment can exert directional selection toward the chloroquine-sensitive allele CVMNK at codons 72 to 76, detectable as early as day 3. This supports our previous findings in Kenya (8). Future studies should deploy this approach to test for selection on other marker genes of interest, including *pfk13*, *pfcoronin*, *pfubp1*, and *pfap2mu* (32).

In the context of Africa, where many participants in clinical malaria studies are children, the utilization of filter paper DNA sampling of finger prick blood taken once daily for the first 3 days of antimalarial treatment is an attractive alternative to frequent (every 6 to 12 h) sampling for microscopy as used for clinical studies of artemisinin efficacy in the Mekong region (7, 18, 19, 21). Although the relatively sparse sampling density makes it more difficult to estimate the slope of the clearance curve accurately, in our hands day 3 qPCR positivity has now proved to be a useful measure of persistent parasitemia, immediately posttreatment, in unrelated studies in Kenya (8), Indonesia (33) and now the West African Sahel. In particular, the relationship between detection of *Plasmodium* DNA at day 3 and subsequent microscopically detectable asexual parasite recurrence in both African studies suggests that our definitions of treatment success and treatment failure need to be reconsidered. The advent of sensitive and precise methods for parasite DNA measurement and quantitation is an opportunity to set a higher bar for treatment success; indeed, it is ironic that whereas DNA amplification methods have been wholly embraced by the malaria research field for determination of PCR-corrected estimates of clinical drug efficacy (34, 35), parasite DNA detection has not been embraced as a primary endpoint in itself. In the current decade, when elimination of malaria remains a firm objective in many settings, we suggest that recurrent parasitemia identified by nucleic acid amplification tests (NAAT) in the follow-up of antimalarial drug trials should be explored as a primary endpoint in treatment trials, particularly in low-transmission settings and those approaching elimination. NAAT are well-established as endpoints in vaccine and drug studies using controlled human malaria infections in volunteers (36, 37) and require only a little further work to be field deployable. An additional benefit is the availability of these DNA samples for concurrent analysis of markers of parasite susceptibility, as demonstrated here for *pfcrt* and *pfmdr1*.

There are a number of aspects of our study that may reduce the generalizability of our findings. Most importantly, the subjects of the work described here are a subset of 552 from a larger study of 4,710 individuals (1). These were not randomly selected but rather were a convenience sample from each of the 5 participating sites, selected on the basis of having a full filter paper set available for each of approximately 105 individuals from each site, within externally imposed time restrictions as to the availability of laboratory capacity in London for Malian and Burkinabe personnel to visit and undertake training in DNA extraction and qPCR, followed by the analysis. Therefore, caution is warranted before generalizing these results to the full study, to the wider West African context, or to the continent in general. Further, there do seem to be differences between sites in the age distributions and the gender ratios (Table 1). More importantly, in one site, Sotuba, a much smaller proportion of individuals with a full set of episode 2 blood samples was included in the analysis, reducing generalizability of our findings regarding time to episode 2. This is probably due to differences in approach to selection of the sample for each local research team. This analysis was not among the primary or secondary outcomes of the main trial, and few resources were available in the field to supervise this aspect. Future studies should seek to perform this analysis for all randomized participants, in order to overcome this problem of potential selection bias, and to clearly set out in the primary protocol all aspects of the procedure.

By current accepted definitions, Sagara and colleagues found AL to have acceptable efficacy in the parent study and that the investigational regimens PA and DP were noninferior and with a good safety profile when tested in a retreatment design over 2 years of follow-up (3). Nevertheless, as discussed in the introduction, these authors presented ample evidence to suggest that these regimens were actually superior to AL in a number of aspects, including ACPR without PCR correction (Fig. 2 in reference 1). As we have provided evidence of a link between qPCR-detectable parasitemia at day 3 and uncorrected ACPR in the AL treatment group, suggesting that persistent parasites in some participants were misclassified by the PCR correction methodology used in WANECAM (1), this may be an important indication that *in vivo* parasite susceptibility to AL is waning in Africa. Our Kenyan studies (7, 8) and identification of treatment failures among AL-treated European travelers add further weight to this interpretation (9, 10). Only a few reports from the field indicate unsatisfactory effectiveness of AL in the African context (5, 6). This could mean that the problem is not yet of public health significance or that in settings with a high proportion of malaria infections occurring in people with some level of previously acquired antiparasite immunity, treatment failure is less easy to identify. Our current data do provide some comfort in this regard: slow clearance in episode 1 was not associated with poor treatment outcomes in episode 2, suggesting that patients were not harboring stubbornly drug-resistant parasite populations that persisted until the subsequent episode. Important questions remain, and we recommend that careful investigations of ACT efficacy using nucleic acid detection as an endpoint be prioritized, at least in low-transmission and/or pre-elimination settings. In addition, a simple surveillance protocol based on a few well-timed finger prick samples linked to national or regional laboratories equipped for DNA extraction and qPCR detection of parasitemia should be on the agenda as a replacement for the current workhorse of blood film microscopy, which nevertheless retains its usefulness for primary diagnosis in African health systems.

In conclusion, the combination of a simple daily finger prick sampling regimen and a well-established qPCR method for measuring parasite clearance dynamics, which is readily transferable (38), has permitted us to show that a relationship exists between persisting submicroscopic parasitemia at day 3 after ACT and microscopically detected treatment failure by day 42. This suggests that the efficacy of AL in particular may be under threat in West Africa, after 10 to 15 years of deployment, and that nucleic acid testing methods may provide a more effective definition of endpoints in future studies of drug efficacy.

## MATERIALS AND METHODS

### Clinical trial design and registration.

This work was performed as an exploratory analysis, defined in the main protocol, of the WANECAM study (1). Trial design, registration, and regulatory approvals are as described therein.

### Patients and study sites.

Research staff at each of three sites in Mali—Sotuba, Kolle, and Bougoula Hameau—and two in Burkina Faso—Niangoloko and Bobo Dioulasso—identified a group of at least 100 WANECAM study participants for which filter paper blood spots were available from each of day 0 (pretreatment) and day 1, day 2, and day 3 of posttreatment follow-up for the primary episode of falciparum malaria that led to the inclusion of the individual in the study. Blood samples taken at day 0, day 1, day 2, and day 3 from individuals with a second episode occurring 28 days or more after the primary malaria episode were also included in the analysis if available.

### qPCR analysis of parasite clearance.

DNA was extracted from filter paper blood samples from each individual using a modification of the Chelex method (39, 40). Parasite DNA at days 0, 1, 2, and 3 was detected using qPCR, and relative parasite density was estimated by amplification of *pgmet* (a genus-specific target) normalized to the human β-tubulin gene *humtubb* as previously described (41). qPCR was performed in duplicate on a Rotor-gene Q thermocycler (Qiagen, Germany). Results were expressed as the parasite reduction rate at 48 h (PRR_48h_) and by the binary outcome positive or negative by qPCR on day 3. This method has now been successfully deployed in a number of field studies in Africa and Asia and has an estimated limit of detection of 0.05 parasite per μl of whole blood (7, 33, 42).

### Genotyping of resistance-associated loci.

Genotyping of the *pfcrt* and *pfmdr1* genes was carried out using established methods, with minor modifications. Polymorphisms at codons 72 to 76 in the *pfcrt* gene were determined using multiplex qPCR (43). Variants at codon 86 of *pfmdr1* were identified by direct sequencing of PCR products as described previously (13, 14).

### Statistical analysis.

Odds ratio (OR) and 95% confidence interval were estimated for each test of association between binary variables, and statistical significance was estimated from the χ^2^ distribution. To evaluate the contribution of variables to day 42 treatment failure and to the time elapsed before the next malaria episode, a logistic regression model was built for each outcome, incorporating all variables found to be significant in univariate analyses, in descending order of OR. Data were tested for normal distribution using the Shapiro-Wilks test, with and without lognormal transformation. Normally distributed continuous data were analyzed using Student’s *t* test and nonnormally distributed data by rank sum test. Parasite density estimates were log transformed for analysis by the rank sum test or for logistic regression. In paired samples, evidence of directional selection was tested for statistical significance by McNemar’s test for symmetry. *P* values of ≤0.05 were considered statistically significant.
